# An Ultra-Low-Power Embedded Processor with Variable Micro-Architecture

**DOI:** 10.3390/mi12030292

**Published:** 2021-03-10

**Authors:** Wenheng Ma, Qiao Cheng, Yudi Gao, Lan Xu, Ningmei Yu

**Affiliations:** Faculty of Automation and Information Engineering, Xi’an University of Technology, Xi’an 710048, China; wenhma@outlook.com (W.M.); 13772525011@163.com (Q.C.); gyd18392005058@163.com (Y.G.); xl314008@163.com (L.X.)

**Keywords:** ultra-low-power, embedded processor, energy efficiency, variable micro-architecture, pipeline register

## Abstract

Embedded processors are widely used in various systems working on different tasks with different workloads. A more complex micro-architecture leads to better peak performance and worse power consumption. Shutting down the units designed for performance enhancement could improve energy efficiency in low-workload scenarios. In this paper, we evaluated the energy distribution in various embedded processors. According to the analysis, pipeline registers and the dynamic branch predictor, which are employed for better peak performance, have great impacts on energy efficiency. Thus, we proposed an ultra-low-power processor with variable micro-architecture. The processor is based on a 4-stage pipeline core with a Gshare branch predictor, and all units work in high-performance mode. In normal mode, the Gshare predictor is shut down and Always-Not-Taken prediction is used. In low-power mode, some of the pipeline registers are bypassed to avoid unnecessary energy dissipation and improve executing efficiency. A mode register (MR) is designed to indicate current working mode. Switching between different modes is controlled by the software. The proposed core is implemented in 40 nm technology and simulated with the traces of 17 benchmarks in Embench. The average amounts of power consumed by the respective modes are 41.7 μW, 59.7 μW and 71.1 μW. The results show that normal mode (N-mode) and low-power mode (L-mode) consume 16.08% and 41.37% less power than high-performance mode (H-mode) on average. In best case scenarios, they could save 25.36% and 49.30% more power than H-mode. Considering the execution efficiency evaluated by instructions per cycle (IPC), the proposed processor consumes 7.78% or 51.57% less energy for each instruction than the baseline core. The area of the proposed processor is only 7.19% larger than the baseline core, and only 3.08% more power is consumed in H-mode.

## 1. Introduction

The Internet of Things (IoT), which excludes PCs, tablets and smartphones, will grow to 25 billion units installed in 2021, representing an almost 30-fold increase from 0.9 billion in 2009 [1]. Additionally, more and more IoT systems are working in battery-powered scenarios or even battery-less scenarios [2,3]. Given the limitation of energy supply and the demand for performance, system designers are paying more attention to power consumption, especially for the embedded processor which is one of the core components in IoT systems [4,5]. Due to its responsibility for data computation and system control, an embedded processor has to deal with a variety of complicated tasks with different workloads, making it difficult to balance peak performance and power consumption for various scenarios. Improving energy efficiency is now becoming a key concern for all designers [6,7].

To satisfy the performance and power demands of diverse embedded systems, processors with different micro-architectures are employed in different devices. For commercial use, ARM Ltd. has introduced Cortex-M series processors, including Cortex-M0, Cortex-M3, etc. [8]. These processors are designed for deterministic, real-time embedded processing and microcontroller applications, and are optimized for low-cost and energy-efficiency. In addition to processors in industry, many cores are implemented in academia [9,10,11,12,13,14]. For example, the Pulp team from ETH and the University of Bologna has proposed several processors, including Zero-riscy, Riscy, and Ariane. All these cores are implemented elaborately for different scenarios, and are silicon-proven for commercial use rather than research-use-only [15,16,17,18,19].

**Figure 1:**
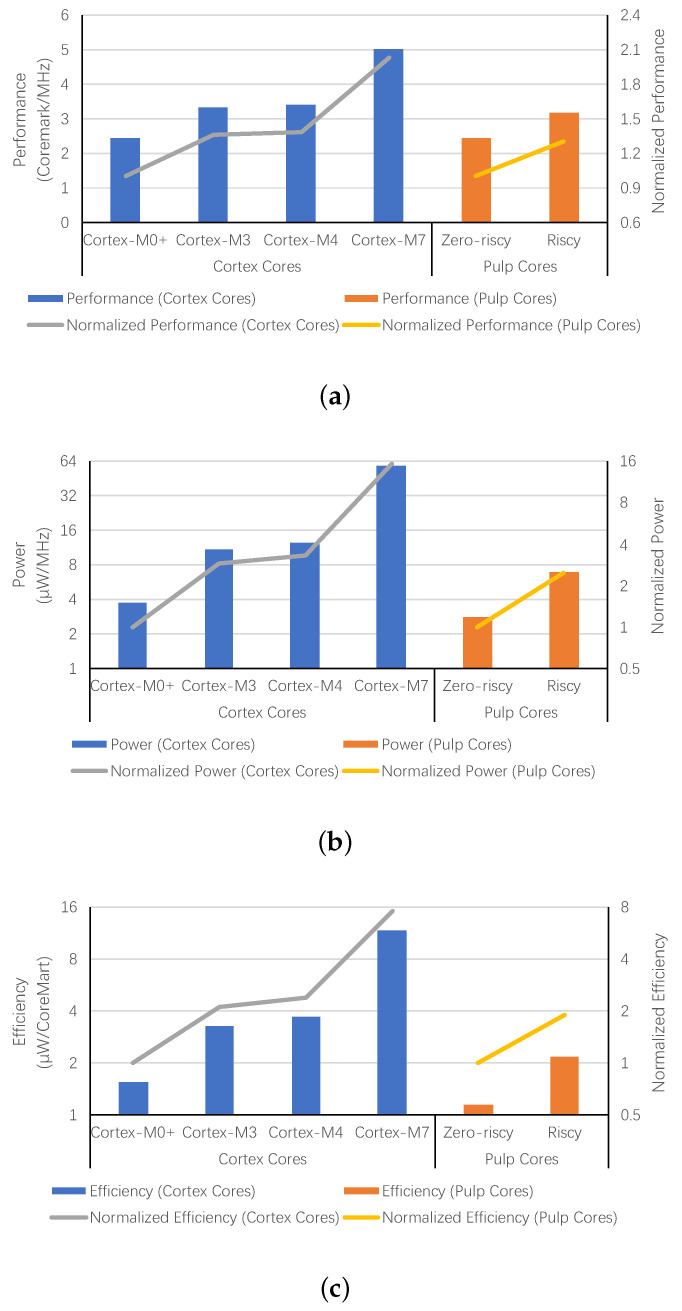
Performance, power and energy efficiency of Cortex-M cores and Pulp cores. (**a**) Performance of Cortex-M Cores and Pulp Cores, (**b**) Power of Cortex-M Cores and Pulp Cores, (**c**) Energy Efficiency of Cortex-M Cores and Pulp Cores.

In many embedded systems, peak performance is a key factor for the processor selection. However, more complex architectures provide better performance and worse power consumption. Despite finishing an instruction in a shorter time, a high-performance processor will still dissipate more energy on the same task and consume more power in idle state. Figure 1 demonstrates the performance, power consumption and energy efficiency of different embedded cores. The performance of Cortex-M3, shown in Figure 1a, is 1.36 times that of Cortex-M0+ [20,21]. However, it consumes 2.89 times the power, which is demonstrated in Figure 1b. According to the results shown in Figure 1c, the tremendous power increase leads to 113% more energy dissipation to execute the CoreMark benchmark once. As the performance and power increase, the energy efficiency for Cortex-M4 and Cortex-M7 is even worse [22,23]. Compared to Cortex-M0+, they use 138% and 656% more energy for the same task. Besides the Cortex-M series cores, Pulp cores are also evaluated in Figure 1. The performance of Riscy is better than Zero-riscy. However, Zero-riscy consumes less energy for a task due to its simpler micro-architecture. For power reduction, scaling down the supply voltage is the most widely used method [24,25]. Some processors are designed to work at a low voltage, or even work at near-threshold or sub-threshold voltage. However, the voltage scaling has slowed down in recent years, since it is no longer possible to scale the threshold voltage because of rising leakage currents [26]. Thus, architecture optimization is now necessary to improve energy efficiency further.

The main focus of this paper is designing an embedded processor with variable architecture. The variable core could change its pipeline structure and shut down the branch predictor to reduce power consumption. The main contributions of this paper are as follows:

1. We present a detailed analysis of the energy distribution of different embedded processors and point out the inefficiency in a processor based on four baseline cores with state-of-the-art performance and power efficiency.
2. We propose a variable processor which could work with different micro-architectures in different working modes.
3. We present a software-based mode switching method that simplifies the circuit design and reduces the hardware overhead.
4. We show the possibility to improve the energy efficiency of an embedded processor by adding simple bypass data paths for architecture switching.

The rest of this paper is structured as follows: Related works are discussed in Section 2. Section 3 presents the baseline processors and analyzes the power distribution in these processors. Section 4 introduces the hardware architecture and software interface of the variable processor. Results and evaluations are shown in Section 5. Section 6 concludes this paper.

## 2. Related Work

In many cases, processors only work on compute-intensive tasks for short periods of time, and on low-load tasks most of the time. To improve the energy efficiency, numerous studies are presented for performance and power consideration [27,28,29,30]. Both circuit-level and architecture-level optimizations were involved in these studies. According to their methods, all the designs can be categorized into (1) systems with dynamic voltage and frequency scaling, (2) heterogeneous multi-core processors and (3) fine-grained heterogeneous processors.

### 2.1. Dynamic Voltage and Frequency Scaling (DVFS)

The voltage of the power supply has a significant impact on energy dissipation. Many digital systems are designed based on a low voltage supply for power reduction. However, a fixed low voltage of the power supply is harmful to the peak performance. To improve energy efficiency and meet the performance demand, dynamic voltage and frequency scaling (DVFS) is widely used, especially for IoT systems [31,32]. By regulating the supply voltage and clock frequency dynamically, a system could manage its performance and power according to the working status. As one of the core components in digital systems, processors benefit greatly from DVFS in low-load conditions as well [33,34]. Besides, DVFS could easily be incorporated into a processor with architecture-level optimization. However, the voltage scaling has slowed down in recent years, since it became impossible to scale the threshold voltage because of rising leakage currents [26]. Considering the limitations of voltage scaling, the utility of DVFS has been decreasing, making it difficult to reduce power dissipation further without architecture optimization.

### 2.2. Heterogeneous Multi-Core Processors

Many works have focused on exploring heterogeneity in a digital system to improve efficiency. Annavaram et al. proposed to use small cores for parallel tasks, while serial tasks are run on a big core [35]. Considering the power limitation, it will improve the overall energy efficiency. Kumar et al. advocated assigning tasks to different cores with various power and performance characteristics, so that each core could achieve the best power efficiency [28,29]. Besides the asymmetric multicore processors, ARM Ltd. has proposed big.LITTLE technology, which contains a big core (Cortex-A15) and a little core (Cortex-A7) in a cluster and only activates one core at a time. Thread switches between cores in coarse granularity. For the same task, the energy efficiency of little cores is much better than that of big cores [36]. To further improve the execution efficiency of these processors, many studies about task mapping and thread switching were also conducted [37,38,39].

### 2.3. Fine-Grained Heterogeneous Processors

Heterogeneous multi-core processors improve performance within a limited power budget, but also lead to additional costs. To avoid the significant drawbacks in terms of area and latency, researchers suggested achieving heterogeneity within a single core. Andrew Lukefahr et al. proposed a composite core with a big μEngine and a small μEngine. The two μEngines share an L1-cache, a branch predictor and an architectural register file. In the composite core, thread switching is controlled dynamically by an online controller [40]. Sudarshan Srinivasan et al. designed a processor that could work in different modes with different micro-architecture resources, e.g., fetch width, issue width and buffer sizes. The processor will perform a program in an appropriate mode determined by a runtime mechanism and switch to another mode when the workload has been changed [41].

Previous works focused on complex heterogeneous processors using an Out-of-Order core as the big core. However, they are too heavy to work in ultra-low-power scenarios. Considering the power limitation, in-order cores without multiple issue ports are always preferred.

## 3. Power Consumption in Embedded Processors

**Figure 2:**
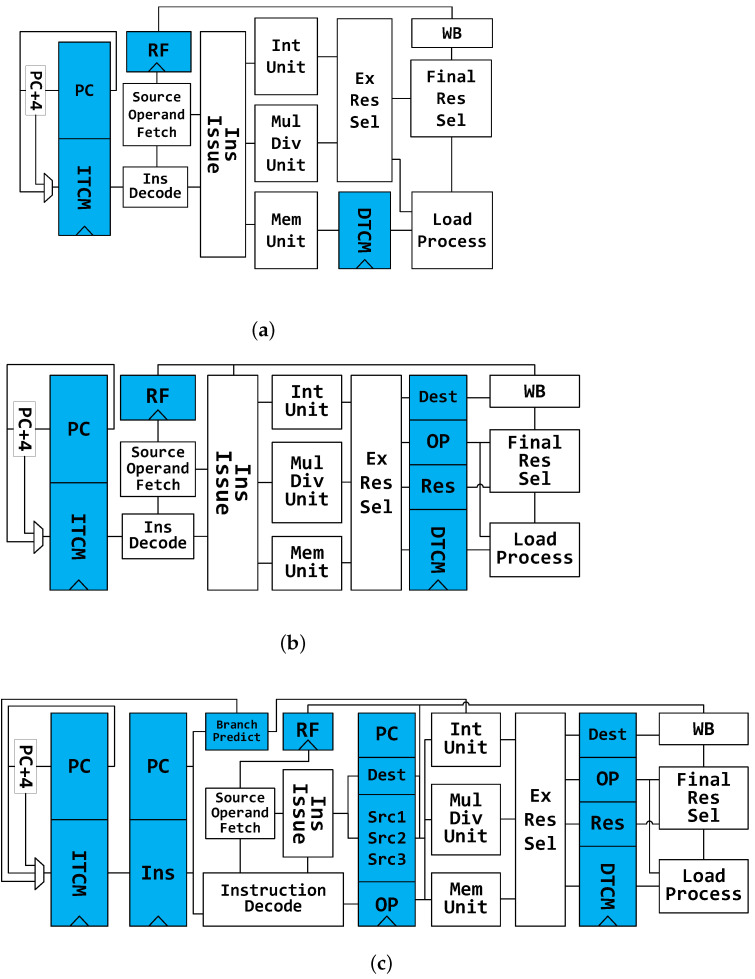
Architecture of baseline processors. (**a**) Architecture of TinyCore, (**b**) Architecture of LittleCore, (**c**) Architecture of PipeCore.

Analyzing the power impact of each part in processors makes sense in order to identify the inefficient units in idle state. To explore the power distribution, we have designed four RISC-V cores supporting RVC32IM and found out the main contributor to energy dissipation. Instructions and data are stored in separate SRAMs. All decoding and execution units are the same in these processors. Thus, the distinction in energy efficiency is mainly caused by different structures, such as control units, branch predictors, pipeline stages, etc. These processors were designed in three different pipeline structures:

1. TinyCore. The most simplified architecture is employed in this core. As is shown in Figure 2a, there is no pipeline register in TinyCore. Only load, multiplication and division instructions can cause stalls in this processor.
2. LittleCore. It has pipeline registers before the Write-Back unit. Figure 2b demonstrates its architecture. Only multi-cycle instructions will stall this processor.
3. PipeCore. The other two processors have the same architecture as the PipeCore shown in Figure 2c. They are both single-issue in-order cores with four pipeline stages and a branch predictor. The only difference between them is the branch prediction algorithm.

Always-Not-Taken and Gshare are used, respectively, in PipeCoreA and PipeCoreG. Gshare is only a tiny predictor without branch target buffer (BTB) and return address stack (RAS) due to their huge requirements in terms of area and energy consumption. Thus, all branch predictions are completed in Decode, which can get the branch target from the instruction. The Gshare predictor has 256 entries. That number of entries was selected because of its better balance between performance and energy efficiency. The maximum frequencies of the cores are 290 MHz (TinyCore), 310 MHz (LittleCore), 740 MHz (PipeCoreA) and 720 MHz (PipeCoreG).

The area, performance and power consumption of these baseline processors are shown in Table 1. All features are close to those of the state-of-the-art cores in academia and industry [8,42]. To further explore the energy efficiency and power distribution of different cores, we have synthesized all these processors with UMC 40LP technology, and evaluated the performance and energy efficiency with the trace of 17 benchmarks in Embench [43]. All cores were synthesized with relaxed timing constraints and run at the frequency of 20 MHz. The execution efficiency of each core was evaluated by the number of instructions per cycle (IPC).

**Figure 3:**
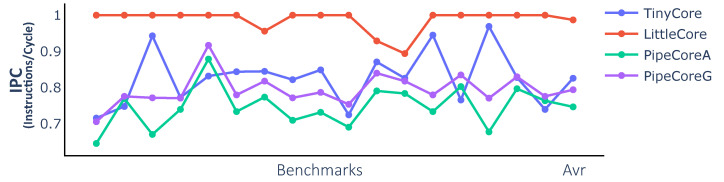
Instructions per cycle (IPC) of baseline Processors.

As shown in Figure 3, there are wide variations in execution efficiency. The IPC values of PipeCores are lower than those of TinyCore and LittleCore. This reduction is caused by the more complex pipeline structure, leading to additional speculative execution and data hazard stalls in the processor. The IPC of PipeCoreG is better than PipeCoreA because of the Gshare predictor. LittleCore has a better IPC than TinyCore, since all load-conflicts are avoided by splitting the load operation into two different pipeline stages and forwarding the result before writing back. Considering the maximum frequency, PipeCoreG offers the best performance followed by PipeCoreA, LittleCore and TinyCore.

**Figure 4:**
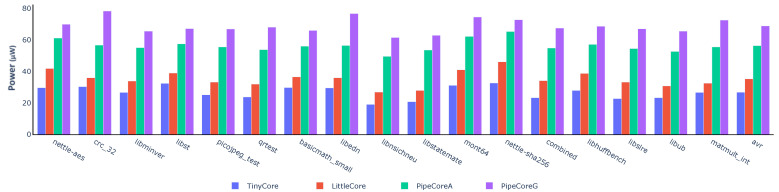
Power consumption of baseline processors.

**Figure 5:**
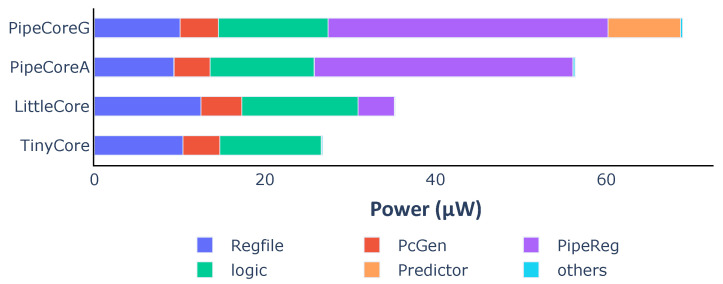
Average power consumption and distribution of baseline processors.

Figure 4 shows the power consumption for each core. Compared to the TinyCore, the other three processors consume 1.32× (LittleCore), 2.11× (PipeCoreA) and 2.58× (PipeCoreG) more power, respectively, on average. Figure 5 demonstrates the power distribution of all baseline processors. The “logic” part in Figure 5 represents the power consumption of all necessary combinational circuits, including instruction decoder, execute unit, load data selector, etc. Since the circuits of the “logic” part are essential and their power consumptions are nearly the same in different cores, we will skip that in the following discussion. Note that the RegFile of LittleCore consumes more energy than others, since its better IPC results in more data writing back within the same time frame. Similarly, more power consumption is needed by the RegFile of TinyCore than that of PipeCores.

**Figure 6:**
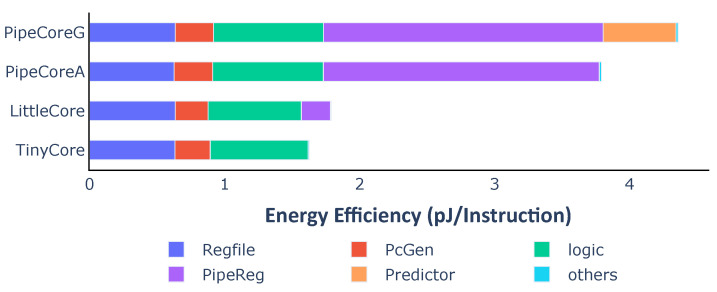
Average energy consumption and distribution of baseline processors.

Taking into account the execution efficiency, the distribution of energy efficiency is a bit different from the power consumption. Figure 6 shows the energy dissipation of each part in a processor for executing one instruction. RegFiles almost consume the same energy in different cores because of the same number of write-backs. PcGenerators and logic circuits in PipeCores consume a little more energy than TinyCore and LittleCore due to the speculative execution. Pipeline registers and the branch predictor have great impacts on energy efficiency. They lead to differences in performance and efficiency among baseline processors.

- Pipeline registers. They are not the key contributor to the area, but consume the most energy than other parts in a processor. For example, the area of the pipeline registers is 1.60 KGE that is only 7.54% of the total core in PipeCoreA. However, they need about 1.96 μW/MHz. That is, over 53.9% of total energy (3.63 μW/MHz) is consumed by them. The counterintuitive result is caused by their higher transition frequency.
- Branch predictor. The dynamic branch predictor has tremendous impacts on performance, power and energy efficiency. The IPC of PipeCoreG is 0.793 which is 6.34% more than that of PipeCoreA. When running at 20 MHz, the power of PipeCoreG is 68.97 μW. That is 22.38% more than PipeCoreA which consumes 56.36 μW. Thus, the Gshare predictor leads to about 15% more energy dissipation for each instruction.

Considering the performance requirements in embedded systems, a deeper pipeline and dynamic branch predictor are indispensable. However, in low-load scenarios, they become a burden on power. Shutting down these units, when they are not necessary in idle state, will be beneficial for energy efficiency.

## 4. Architecture and Implementation

### 4.1. Multiple Operating Modes

Performance and power differ greatly among these baseline processors. An aggressive branch predictor and pipeline structure, which are not essential to complete an instruction, are the main contributors to power increasing. Working without these units will improve energy efficiency. Thus, the ability to run in diverse operating modes is useful for an ultra-low-power processor to balance the performance and energy efficiency.

In the previous section, four baseline cores were discussed. It is not a good idea to support all modes in the design, since more modes may lead to an unnecessary overhead. Among these cores, PipeCoreG has the best performance that is important for many applications. Additionally, the only difference between PipeCoreA and PipeCoreG is the branch predictor. Shutting down the Gshare predictor by inserting several clock gating cells makes the PipeCoreG working like PipeCoreA. Because of the negligible cost, their micro-architectures are both selected as two different modes. The performance of TinyCore is much worse than LittleCore with similar energy efficiency. Therefore, supporting LittleCore only is preferred rather than having two individual modes for them, since additional MUXes are needed for the architecture of TinyCore.

Taking all factors into account, there are three operating modes in the variable architecture processor: (1) High-Performance mode (H-mode). The processor works with a 4-stage pipeline and a Gshare branch predictor. (2) Normal mode (N-mode). The 4-stage pipeline is still employed while shutting down the Gshare predictor. (3) Low-power mode (L-mode). Pipeline registers between Fetch and Execute are all bypassed and no speculative structure is used in this mode. Switching between different modes is controlled by software.

### 4.2. Variable Branch Predictor

**Figure 7:**
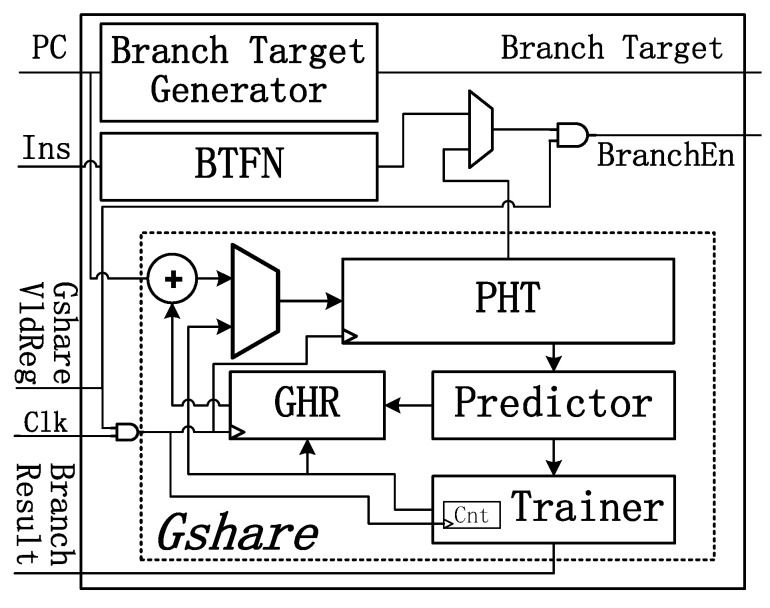
Variable branch predictor.

Figure 7 shows the architecture of the variable branch predictor. A memory-mapped register named GshareVldReg is employed in the processor. The branch predictor could work in two different modes indicated by the GshareVldReg.

If GshareVldReg is 1, the prediction result of Gshare is enabled. Prediction is performed in the Decode unit, and the branch target is calculated from the instruction instead of using a branch target buffer (BTB) to avoid additional energy costs. The saturating counter in the Gshare predictor will be out of service when training the pattern history table (PHT), since the PHT SRAM has only one port. Thus, Backward-Taken-Forward-Nottaken(BTFN) prediction is used if a training conflict appears.

When GshareVldReg stays at 0, the Always-Not-Taken algorithm is used. To reduce the energy consumption, the clock to Gshare is gated, and operand isolation is used in the branch target generator. Thus, all the logic circuits, registers and SRAMs keep silent until the operating mode has been changed. In this way, most of the energy could be saved due to the shutting down of Gshare predictor. Only several AND gates are inserted into logic circuits, leading to little costs in terms of area. As these additional gates are not on the critical path, there is no timing overhead for them.

### 4.3. Variable Pipeline Architecture

**Figure 8:**
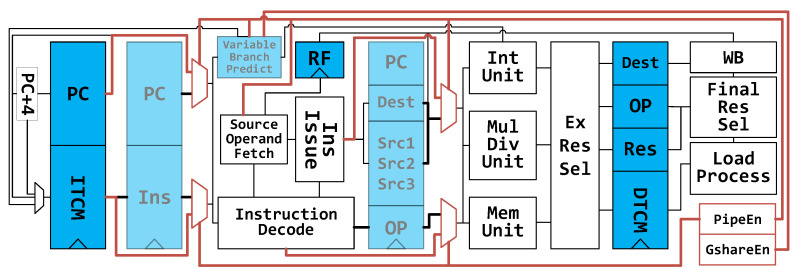
Variable architecture. Pipeline registers and branch predictor can be disabled in normal mode (N-mode) and low-power mode (L-mode). They are translucent in the figure.

**Figure 9:**
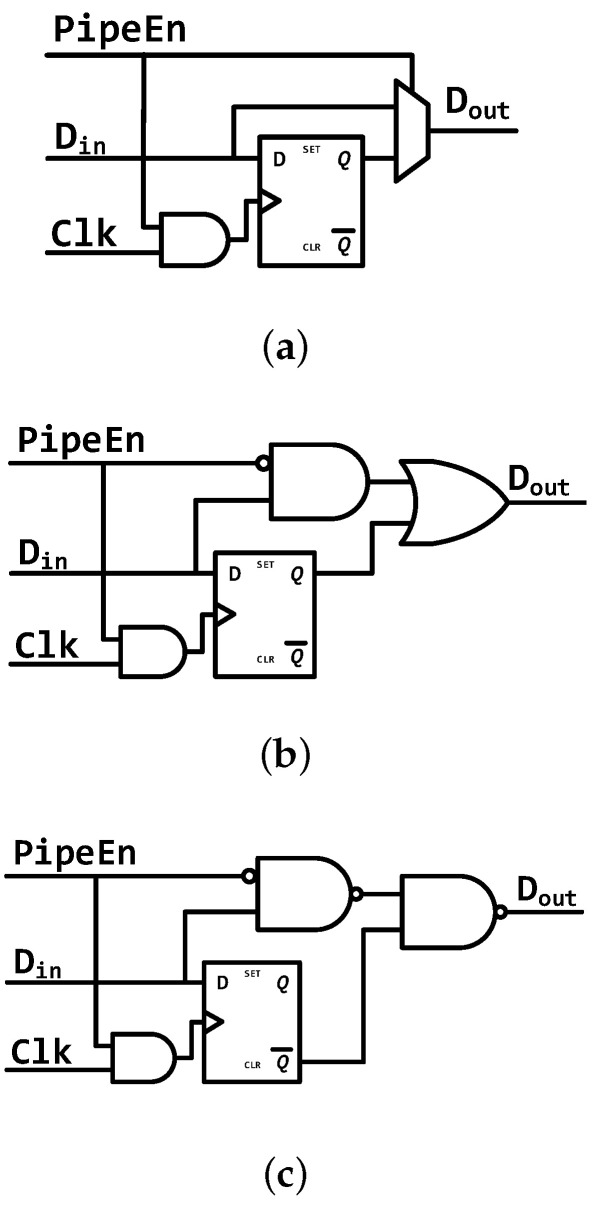
Bypass circuits for each pipeline register. (**a**) MUX Bypass, (**b**) AND-OR Bypass, (**c**) NAND-NAND Bypass.

Figure 8 demonstrates the variable-architecture processor. It is based on the PipeCore shown in Figure 2c. To eliminate the wasted energy in pipeline registers, all registers between Fetch and Execute are bypassed, making the core work with a 2-stage pipeline, similarly to the LittleCore mentioned in Figure 2b. All bypass transmission is achieved by inserting MUXes after pipeline registers, as shown in Figure 8 and Figure 9a. To simplify the MUX circuits, all pipeline registers are flushed to zero before clock gating. Hence, a real multiplexer is not necessary for signal forwarding. We can use an AND-OR gate, as shown in Figure 9b, instead of a multiplexer. That will reduce the size by one AND gate. Additionally, the AND-OR gate also can be simplified to a NAND-NAND gate by inverting the output of each pipeline register, as shown in Figure 9c. As a result, only 8 transistors are needed to bypass a signal. That is fewer than a multiplexer (12 transistors) or an AND-OR gate (10 transistors).

A forward bus is used in the 4-stage pipeline architecture to access data before writing them to the register file. In the variable architecture, the destinations of forwarding data come from the pipeline registers directly. In low-power mode, the forwarding data from Execute is invalid, since the pipeline register between Decode and Execute is bypassed, and all bits in the register are flushed to 0. Thus, the destination keeps 5${}^{\prime }$b00000 and the forward bus will be out of service.

In addition to the data path, the execution controlling mechanism in low-power mode is also different. There are no pipeline stalls caused by data hazards in this mode. The controller only stalls the processor when executing multiplication and division, which could take additional cycles. Another memory-mapped register is used to indicate whether the processor is working with or without a complex pipeline structure. The register is named PipeEn, as shown in Figure 8. When writing 0 to this bit, the processor flushes all pipeline registers between Fetch and Execute, and then blocks the clock to those pipeline registers.

### 4.4. Software-Based Mode Switching

Mode switching is controlled by software. There is a mode register (MR) in the processor indicating current operating mode. PipeEn assigned to bit[1] and GshareEn assigned to bit[0] are used to indicate the pipeline architecture and the type of branch predictor. Note that, there are only three modes in the processor, and therefore writing 2${}^{\prime }$b01 to the mode register is invalid. When the processor switches from L-mode to N-mode or H-mode, the following steps must be done in software:

1. Set mode register to 2${}^{\prime }$b1x (2${}^{\prime }$b10 for N-mode; 2${}^{\prime }$b11 for H-mode).
2. Increase the clock frequency.
3. Jump to compute-intensive tasks.

When switching to L-mode, the following things must be done by software to change the micro-architecture:

1. Reduce the clock frequency.
2. Set MR to 2${}^{\prime }$b00.
3. Execute two NOP instructions.
4. Jump to low-load tasks.

**Figure 10:**
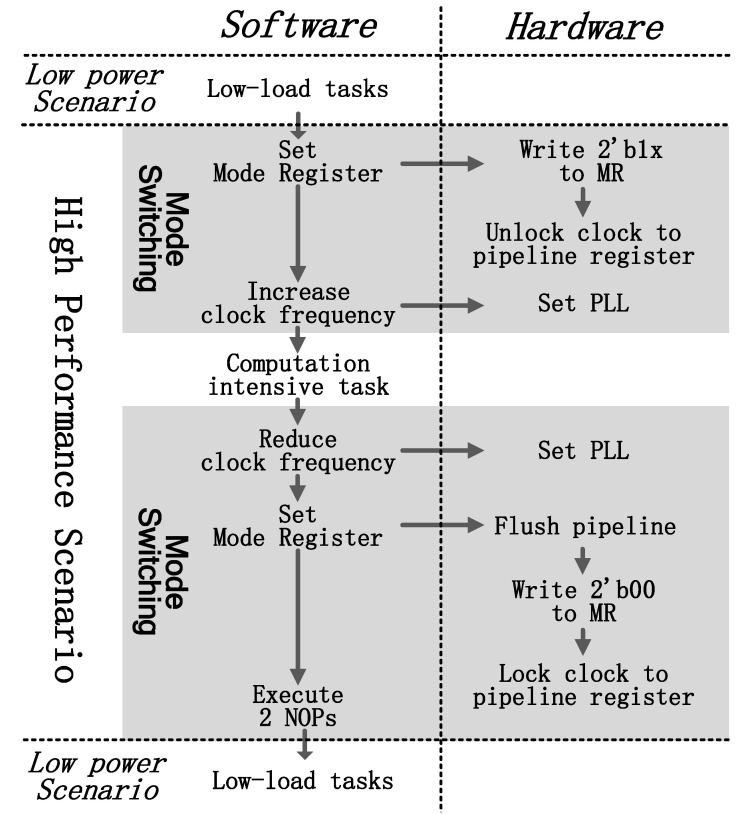
Software-based mode switching flow.

Two extra NOPs are inserted after setting the MR. There are three instructions executing in the pipeline simultaneously besides the MR setting instruction, and the flushing pipeline registers will kill the following two instructions. An example of switching flow is shown in Figure 10. The processor works in L-mode for low-load tasks at first. When a compute-intensive task is coming, MR is set to 2${}^{\prime }$b1x in the software. The processor will flush the pipeline registers and unlock their clock. Then it increases the clock frequency and works on the coming tasks. When the tasks are finished, the processor reduces the clock frequency and writes 2${}^{\prime }$b00 to MR turning to L-mode.

The variable branch predictor could change the prediction algorithm in one cycle and lock or unlock the Gshare predictor next cycle. Switching between H-mode and N-mode only needs two steps in software:

1. Write corresponding data to GshareEn register (bit[0] in MR).
2. Jump to new tasks.

## 5. Experiment Results and Discussion

We have implemented the variable core in UMC 40LP. It has 26.59 K gates, which is only 7.19% more than PipeCoreG. Additionally, its maximum frequency is 2.3% slower due to the additional NAND cells in the critical path. In low power mode, the maximum frequency is about 260 Mhz. The micro-architectures of the variable core in different operating modes are the same as the corresponding baseline processors. Thus, they have the same execution efficiency evaluated by IPC.

**Figure 11:**
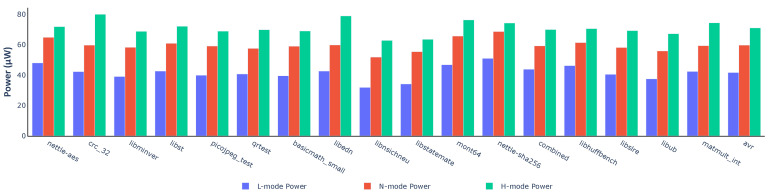
Power consumption of the variable processor in different modes.

First, we simulated the processor in different modes with a clock frequency of 20 MHz. Figure 11 demonstrates the power consumption of each benchmark in Embench. In all cases, working in L-mode reduces power consumption greatly. N-mode also needs less power than H-mode. Compared with H-mode, N-mode and L-mode could save more than 25.36% and 49.30% power in the best cases. Even in the worst cases, about 7.54% and 31.40% power are saved.

**Figure 12:**
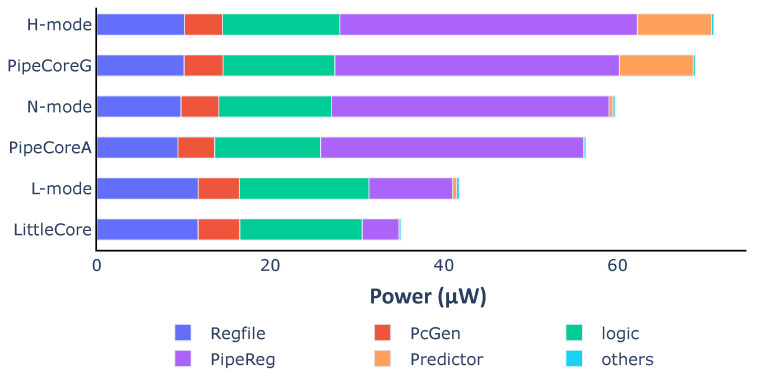
Average power consumption and distribution of the variable processor in different modes.

As shown in Figure 12, the average power of different modes are 41.7 μW, 59.7 μW and 71.1 μW, respectively. Typically, N-mode and L-mode consume 16.08% and 41.37% less power than H-mode within the same clock period. The power consumption of each mode increases a little over the value that is in the corresponding baseline core. This is caused by two reasons: (1) The execution controller in the variable core is a little more complicated than those of TinyCore and LittleCore. Thus, the power of the “logic” part increases a little. (2) Many NAND cells are inserted after some of the pipeline registers, leading to the power increasing in the “PipeReg” part.

**Figure 13:**
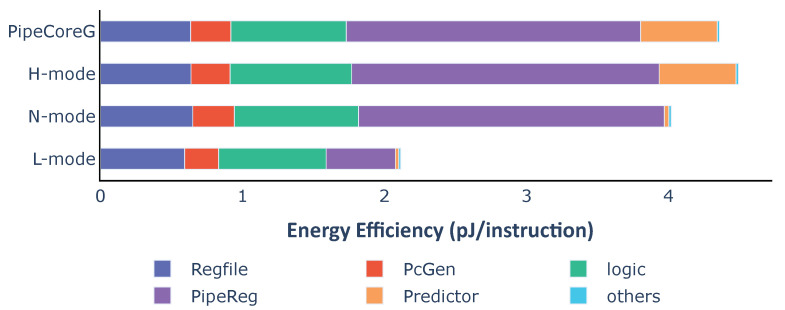
Average energy consumption and distribution of the variable processor in different modes.

Energy dissipation for each instruction is shown in Figure 13. To execute one instruction, the variable core in different modes consumes 2.12 pJ (L-mode), 4.02 pJ (N-mode) and 4.50 pJ (H-mode), respectively. For each instruction, the variable core in L-mode needs 52.92% less energy than H-mode. Compared with PipeCoreG consuming 4.36 pJ/instruction, the variable core could save 7.78% and 51.57% more energy in N-mode and L-mode. Only 3.08% more power is consumed in H-mode. According to the power and energy distribution, shutting down the branch predictor saves 0.509 pJ energy which is 92.2% of total energy consumed by the Gshare predictor in PipeCoreG. Bypassing pipeline registers between Fetch and Execute saves 1.585 pJ energy. That is 76.48% of all pipeline registers in PipeCoreG. The energy overhead of H-mode, which is 0.134 pJ, is 3.08% of the total power in PipeCoreG. Most of the additional energy is caused by MUX logics after pipeline registers.

Table 2 shows the peak performance with tight timing constraints and the energy efficiency with relaxed timing constraints for our proposed processor and the state-of-the-art cores. In view of the maximum frequency, the peak performance of the variable core in H-mode and N-mode is better than riscy and Cortex-M4. Additionally, its peak performance in H-mode is a little better than in N-mode due to a better branch predictor. For low-load scenarios, the variable core, working in L-mode, needs less power than zero-riscy and Cortex-M0+. Therefore, the variable core provides a better trade-off between peak performance and energy efficiency, even if the switching circuit involves some power overhead.

**Figure 14:**
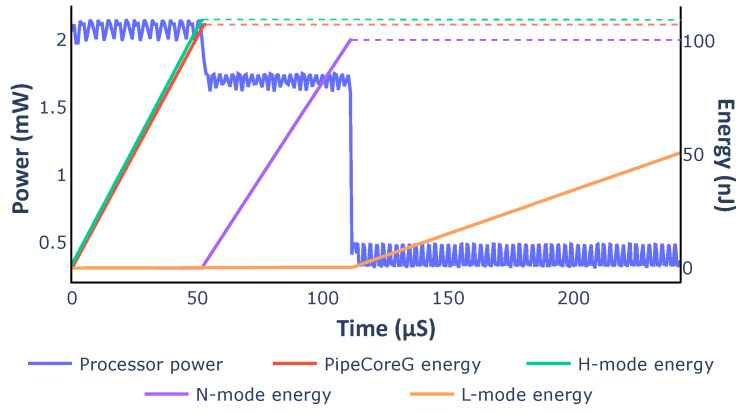
Dynamic power and energy for DMIPS.

We simulated the variable core with dynamic mode switching. The core executed the Dhrystone benchmark (DMIPS) 50 times in each mode. Energy consumption was calculated by integrating the corresponding power curve. All curves are demonstrated in Figure 14. The energy curve for PipeCoreG running DMIPS 50 times is also shown in Figure 14 as a reference. The variable core works at 200 MHz in L-mode, while the other modes and PipeCoreG work at 600 MHz. As the result shows, complicated micro-architectures lead to better peak performance, reducing the execution time for the same task. Simpler micro-architectures improve energy efficiency greatly. Embedded systems could benefit greatly from the variable architecture, if they work on low-load tasks most of the time and handle compute-intensive tasks for only short periods.

## 6. Conclusions

This paper has analyzed the power distributions of embedded processors with different architectures, and pointed out the key factors reducing energy efficiency. As the results show, the pipeline registers consume 53.9% power with only 7.54% of the total area, and the branch predictor leads to 22.38% more power dissipation with an only 6.34% performance increase. To improve energy efficiency in idle state, a variable processor with three operating modes was proposed in this paper. The core is a 4-stage pipeline processor with a Gshare predictor in high-performance mode. It could shut down unnecessary units in different modes to improve energy efficiency. In normal mode, an Always-Not-Taken predictor is used instead of a Gshare predictor. Additionally, some of the pipeline registers are bypassed in low-power mode.

The variable core, in N-mode and L-mode, consumes 16.08% and 41.37% less power than H-mode on average. Compared with the baseline processor, the proposed processor needs 7.78% and 51.57% less energy to execute one instruction in N-mode and L-mode, respectively. In H-mode, only 3.08% more power is consumed in our design, with 7.19% more gates being involved. The result illustrates that processors with the variable architecture and multiple operating modes will achieve better energy efficiency in various scenarios and meet the demands of peak performance.

For complex tasks in embedded systems, more complicated architectures are employed, such as multi-issue and out-of-order, due to the increase in performance demand. These processors may be simplified by adding new data paths to support architecture switching. For further exploration, our proposed software-controlled variable architecture may also be applied to these complicated processors to balance peak performance and energy efficiency.

## Figures and Tables

**Table 1:** Comparison of baseline processors and the state-of-the-art cores.

Core Name	Pipe No.	Hard Mul Div	Technology	Power Supply	Area (KGE)	Perf (CoreMark/MHz)	Power (W/MHz)
TinyCore	/	Y	40 nm	1.1 V	19.3	2.88	2.12
LittleCore	2	Y	40 nm	1.1 V	19.8	3.44	2.29
PipeCoreA	4	Y	40 nm	1.1 V	21.2	2.75	3.63
PipeCoreG	4	Y	40 nm	1.1 V	24.8	2.91	5.32
micro-riscy	2	N	65 nm	1.2 V	11.6	0.91	2.33
zero-riscy	2	Y	65 nm	1.2 V	18.9	2.44	2.81
riscy	4	Y	65 nm	1.2 V	40.7	3.19	6.98
Cortex-M0+	2	Y	40 nm	1.1 V	12.5	2.46	3.8
Cortex-M3	3	Y	40 nm	1.1 V	37.9	3.34	11
Cortex-M4	3	Y	40 nm	1.1 V	53	3.42	12.26

**Table 2:** Comparison with the state-of-the-art processors.

Core Name	Technology	Power Supply	Area (KGE)	Performance (CoreMark/MHz)	Max Frequency (MHz)	Power (μW/MHz)	Peak Performance (CoreMark/s)	Efficiency (μW/CoreMark)
Variable Core(L-mode)	40 nm	1.1 V	25.9	3.44	260	3.12	894	0.91
Variable Core(N-mode)	40 nm	1.1 V	25.9	2.75	700	4.50	1925	1.64
Variable Core(H-mode)	40 nm	1.1 V	25.9	2.91	700	5.49	2037	1.89
PipeCoreG	40 nm	1.1 V	24.8	2.91	720	5.32	2095	1.83
zero-riscy [42]	65 nm	1.2 V	18.9	2.44	560	2.81	1366	1.15
riscy [42]	65 nm	1.1 V	40.7	3.19	560	6.98	1786	2.19
Cortex-M0+ [44]	40 nm	1.1 V	12.5	2.46	297	3.8	730	1.54
Cortex-M4 [45]	40 nm	1.1 V	53	3.42	223	12.26	762	3.58
